# Supplementary figures and images for: Suitability of IgG responses to multiple Plasmodium falciparum antigens as markers of transmission intensity and pattern
Source: PLoS One. 2021 Apr 22;16(4):e0249936. doi: 10.1371/journal.pone.0249936 (PMC8062017; doi:10.1371/journal.pone.0249936)

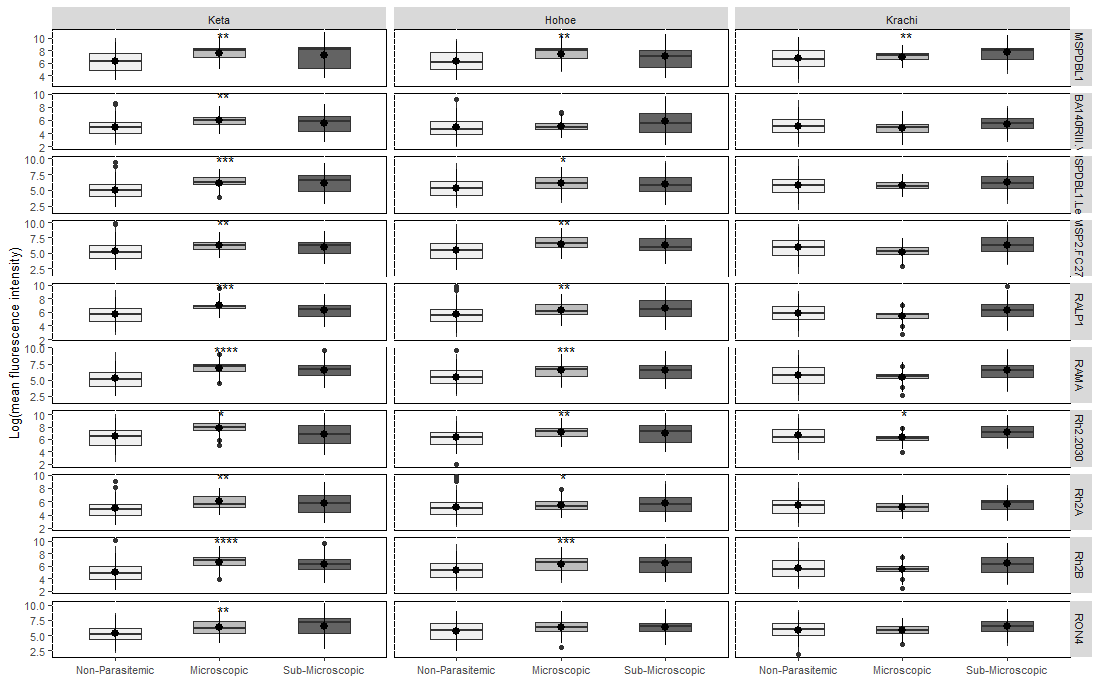

Supplement: S1 Fig — Box and whisker plots with a round dot in the middle, the median IgG level of the group. Differences in antibody levels between non-parasitaemic, microscopic, and sub-microscopic individuals for each antigen are shown for each district. The x-axis represents the districts, and the y-axis represent the log-transformed antibody levels (** p<0.01, ***p<0.001, ****p<0.0001). (TIF) [file pone.0249936.s001.tif]
