# Supplementary material for: Suitability of IgG responses to multiple Plasmodium falciparum antigens as markers of transmission intensity and pattern
Source: PLoS One. 2021 Apr 22;16(4):e0249936. doi: 10.1371/journal.pone.0249936 (PMC8062017; doi:10.1371/journal.pone.0249936)
Supplement: Attachment — Submitted filename: Responses Reviewers 38844R1.docx [file pone.0249936.s003.docx]

**Title of paper: PONE-D-20-38844R1**

Suitability of IgG responses to multiple Plasmodium falciparum antigens as markers of transmission intensity and pattern

***Kyei-Baafour et al.***

Dear Editor,

Thank you for your email on 15 March 2021, in which you informed us that our manuscript after careful consideration, you feel that it has merit but does not fully meet PLOS ONE’s publication criteria as it currently stands. Therefore, you invited us to submit a revised version of the manuscript that addresses the points raised during the review process. (Manuscript ID PONE-D-20-38844R1)), title above.

We thank the Editor and reviewers for their critical review and additional comments which we know when addressed will improve the manuscript.

We hereby submit for your consideration a revised version, in which we have carefully considered all the comments and suggestions made by the reviewer and the editorial team.

Please find below our point-by-point response to editorial corrections and reviewers’ comments, and a tracked changes version of the revised manuscript.

Thank you.

Yours Sincerely,

Michael F. Ofori

**EDITORIAL COMMENT:**

1. Please review your reference list to ensure that it is complete and correct. If you have cited papers that have been retracted, please include the rationale for doing so in the manuscript text, or remove these references and replace them with relevant current references. Any changes to the reference list should be mentioned in the rebuttal letter that accompanies your revised manuscript. If you need to cite a retracted article, indicate the article’s retracted status in the References list and also include a citation and full reference for the retraction notice.

**Response**: ” Reference # 32” (**Aase A, Sandlie I, Norderhaug L, Brekke OH, Michaelsen TE. The extended hinge region of IgG3 is not required for high phagocytic capacity mediated by Fc gamma receptors, but the heavy chains must be disulfide bonded. Eur J Immunol. 1993; 23:1546-51**) on page 7 line 156 of the manuscript has been removed because it was a wrong citation. It was supposed to be **EBA140RIII-V-{32}** and not as **Ref (32)**

We have also added 4 new references (47-49 and 53) as a result of the recommendation by Reviewer #1 to discuss the results on sub-microscopic parasitaemia

**Reviewer #1 Comments**

2. The authors adequately responded to all comments. Inclusion of the sub-microscopic data clearly improves the impact of this manuscript but needs to be well discussed.
   Just some minor comments

   Line 270: 274: Was there any antibody difference between microscopic and submicroscopic groups? Since this data is available it’s should be mentioned as well as discussed for Keta and Hohoe vis a vis Krachi. And how does this affect or influence transmission?, and relate with the authors conclusion that, “These indicate that a threshold of antibody level is needed to control parasitaemia and thus protection”.

**Response:** Generally, there was a trend of higher responses in the sub-microscopic group compared to the microscopic group though not significant across the three sites except MSP2_-FC27_ and Rh2_-2030_ which were statistically high in the sub-microscopic group in Krachi, we have therefore inserted a statement describing these results under the results section (Page 14 lines 276-279) to reflect the new addition. In addition, the relationship between sub-microscopic parasitaemia and transmission has been discussed (Page 21 line 416-422). Slight modifications have been made on page 21 lines 413 -415.

3. Line 309: MSPDBL_Leucine was not consistently revised correctly. Line 302 vs line 285 reads MSPDBL1_Leucine. Generally, all antigen names should be checked again.

**Response:** All antigen names have been corrected throughout the text to be consistent ( Eg MSPDBL_Leucine has been changed to MSPDBL_Leucine,_ in Table 2, **RH22030 to *Pf* Rh2_2030,_ in Table 2 and throughout the text, etc)**
